# In vivo imaging of mitochondrial function in normal, glaucoma suspect, and glaucoma eyes

**DOI:** 10.1371/journal.pone.0317354

**Published:** 2025-01-14

**Authors:** René Caro, Andrew Chen, Raghu Mudumbai, Eric Duerr, Philip P. Chen, Karine D. Bojikian

**Affiliations:** Department of Ophthalmology, University of Washington, Seattle, WA, United States of America; Doheny Eye Institute, UNITED STATES OF AMERICA

## Abstract

To investigate macula and optic nerve head (ONH) mitochondrial metabolic activity using flavoprotein fluorescence (FPF) in normal, glaucoma suspect (GS), and open-angle glaucoma (OAG) eyes we performed a cross-sectional, observational study of FPF in normal, GS, and OAG eyes. The macula and ONH of each eye was scanned and analyzed with a commercially available FPF measuring device (OcuMet Beacon, OcuSciences Inc., Ann Arbor, MI). One-way analysis of variance was used to compare macula and ONH FPF scores between groups. Linear regression models investigated the correlation between FPF scores and structural and functional parameters. We included 25 normal, 16 GS, and 54 OAG eyes. The average age in years ± SD for normal, GS, and OAG groups was 60.6 ±17.4, 67.8 ± 10.3, and 67.9 ± 11.6, respectively (*P* = 0.064). There was no significant difference in gender, race/ethnicity, visual acuity, and intraocular pressure between groups. OAG eyes had larger cup-to-disc ratio, thinner retinal nerve fiber and macula thicknesses, and worse visual field indices compared to normal and GS eyes (*P* ≤ 0.018). There was no significant difference in any FPF metric between the study groups in either the macula or the ONH, despite normalizing FPF data for structural differences between groups (e.g. retinal nerve fiber layer and ganglion cell-inner plexiform layer thickness). In conclusion, no significant differences in metabolic activity as measured by FPF were found in macula and ONH FPF scores using the integrated clinician report generator between normal, GS, and OAG eyes. Further research is needed to evaluate the role of mitochondrial metabolic activity measurements in glaucoma.

## Introduction

Glaucoma is a leading cause of irreversible blindness worldwide [1]. It is characterized by retinal ganglion cell (RGC) degeneration, progressive changes of the optic nerve head (ONH) and retinal nerve fiber layer (RNFL), and associated visual field (VF) loss [2]. The precise pathophysiological mechanism of glaucoma remains under investigation, but elevated intraocular pressure (IOP) is a major risk factor for its development and progression [2, 3]. A prior report showed that patients with open-angle glaucoma (OAG) had higher rates of mitochondrial DNA mutations and decreased mitochondrial respiratory activity compared to age-matched controls [4]. The RGCs and ONH, the major sites of glaucomatous damage, contain a high density of mitochondria, and oxidative stress, mitochondrial dysfunction, and impaired energy metabolism have emerged as potential contributors to the pathophysiology of glaucoma [5–7].

Prior to apoptosis, mitochondria exhibit impaired electron transport by energy-generating enzymes in the respiratory chain, causing increased percentages of flavoproteins in the chain to be oxidized. Mitochondria in this state may absorb blue light (430 to 470 nm) and emit green fluorescence (520 to 540 nm), a phenomenon termed flavoprotein fluorescence (FPF) [8]; FPF could, therefore, be used to identify potential signs of early retinal disease before any significant anatomic alterations occur. Prior studies using a prototype investigational FPF-measuring device reported higher levels of macular FPF in ocular hypertension (OHT) and OAG compared to normal eyes [9], and higher levels of FPF in the peripapillary area in OAG compared to normal eyes [10]. Additionally, antioxidant supplementation has been shown to reduce RGC loss and preserve retinal nerve fiber layer (RNFL) thickness [11]. Still, there is a paucity of data on the use of FPF as a biomarker for glaucoma.

The purpose of this study was to quantify and compare *in vivo* mitochondrial metabolic activity in two regions, the macula and the ONH, in normal, GS, and OAG eyes, using a commercially available FPF measurement system.

## Methods

The Institutional Review Board of the University of Washington (UW) approved the study protocol, and written informed consent was obtained from all participants before imaging. This study followed the tenets of the Declaration of Helsinki and was conducted in compliance with the Health Insurance Portability and Accountability Act.

Subjects with the diagnosis of OAG, GS, or normal optic discs were prospectively enrolled at the UW Medicine Eye Institute from 4/17/2023 to 12/22/2023. The electronic medical records were assessed from 4/17/2023 to 1/21/2024 and reviewed for demographic and clinical exam information, including cataract grade and phakic/pseudophakic status, pertinent systemic comorbidities, and ocular comorbidities. Inclusion criteria included adults age > 18 years old, best-corrected visual acuity (BCVA) of 20/40 or better, and open angles on gonioscopy. We excluded eyes with ocular disease that may impact image acquisition or FPF measurements, including vitreoretinal pathologies such as diabetic retinopathy and macular degeneration; previous intraocular surgeries (although uncomplicated incisional glaucoma surgery, minimally invasive glaucoma surgery, or cataract surgery was allowed, unless surgery occurred within 3 months from study scan); and significant media opacity preventing high-quality imaging.

All subjects underwent a comprehensive ophthalmologic examination by a glaucoma specialist at the time of enrollment, including tonometry by Goldmann applanation, slit lamp biomicroscopy, and fundus examination, and OAG and GS subjects underwent VF and optical coherence tomography (OCT) (Spectralis; Heidelberg Engineering, Germany) testing. For normal eyes, subjects were required to have IOP less than 21 mmHg by Goldmann applanation tonometry and a healthy optic disc on fundoscopic examination, while GS subjects were selected based on the presence of suspicious appearance of the optic disc (neuroretinal rim thinning, excavation, or retinal nerve fiber layer (RNFL) thickness below 95% confidence interval), IOP < 21 mm Hg, and no evidence of reproducible glaucomatous VF damage or progressive RNFL thickness thinning during follow-up. The diagnosis of OAG was based on characteristic optic disc findings, a RNFL thickness on OCT outside the 95% confidence interval; and a corresponding glaucomatous VF loss, such as an isolated scotoma, an arcuate scotoma, a nasal step, a hemifield defect, a generalized depression, or any combination of these, irrespective of IOP.

All VFs were performed on a Humphrey Field Analyzer 3 (Carl Zeiss Meditec, Dublin, CA) using 24–2 Swedish interactive testing algorithm (SITA) Standard or 24-2C SITA Faster strategies, stimulus size III; only reliable tests were included (≤33% fixation losses, false-negative results, and false-positive results). Glaucomatous eyes were divided into 3 severity stages (mild, moderate, and severe) based on the VF mean deviation (MD) [12]. Mild stage included MD ≥ -6.00 dB, moderate stage included MD < -6.00 dB and ≥ -12.00 dB, and severe stage included MD < -12.00 dB.

All subjects had FPF imaging using the OcuMet Beacon (OcuSciences Inc., Ann Arbor, MI). Some patients were dilated if image quality was insufficient (as described below) without dilation in a dark room. The device captures a 60° x 21° IR image and a 17° x 21° FPF image. The light sources include an IR LED (825–870 nm) and a blue LED (458 ± 2 nm); it is classified as a Group 1 device for light safety under American National Standards Institute Z80.36–2016 and International Organization for Standardization 15004 guidelines. The detected fluorescence spectrum is 520 to 540 nm. The images were automatically detected by the device, centered first over the ONH and then on the foveal pit. The quality of images was determined by OcuMet Clinician Report Generator Image Quality Standards, version 0, as follow: high quality (IR images with an index less than 28 and FPF images with an index less than 19), sub-optimal quality (IR images with an index between 28 to 35 and FPF images with an index between 19 to 22), and poor-quality (IR images with an index 35 or over and FPF images with an index 22 or over). Low-quality images were excluded from the analysis, including images that were out of focus, were off-center, contained artifacts, or had sub-optimal pupillary dilation. Low-quality images were expected with pupillary diameter between 2.0 mm and 3.5 mm; of note, the device cannot capture images with pupillary diameter less than 2.0 mm. At least two high-quality images were obtained for each eye. The integrated OcuMet Clinician Report Generator (version 1.206.148) was used to analyze the images: the average FPF intensity is calculated as the mean score from all pixels within a 17° x 21° field, and the FPF heterogeneity is calculated as the standard deviation of FPF intensity.

Since fewer functioning mitochondria are present as glaucoma progresses, and FPF scores could be falsely normal due to fewer mitochondria fluorescing under stress, the average ONH FPF scores were also normalized by global RFNL thickness, while the average macula FPF scores were normalized by the temporal RNFL, total macula, and GCIPL thicknesses.

Statistical analyses were performed using the IBM SPSS Statistics (IBM Corp. IBM SPSS Statistics for Windows, Version 29.0.1.0 Armonk, NY: IBM Corp). The BCVA in Snellen was converted to LogMAR. One eye from each subject was randomly chosen if both eyes were eligible. One-way analyses of variance (ANOVA) was used to assess for any differences in the macula and ONH FPF scores among normal, GS, and OAG eyes. A *P* value < 0.05 was considered statistically significant. Multiple individual comparisons were conducted between pairs of groups via independent, two-sample t-tests. Bonferroni adjustment was applied to keep the overall Type I error maintained at 5%, and therefore, for each individual comparison, p<0.0167 was considered statistically significant. Linear regression models were further used to investigate the correlation between ONH and macula FPF scores, and ganglion cell inner plexiform layer (GCIPL) and RNFL thickness, CDR, and VF indices.

## Results

Twenty-five eyes from normal subjects, 16 eyes from GS subjects, and 54 eyes from OAG subjects were enrolled. Thirty-three (61.1%) OAG eyes had mild glaucoma, 12 (22.2%) had moderate, and 9 (16.7%) had severe based on VF MD. Table 1 summarizes the demographic information and structural clinical measurements. No significant differences were detected in age, race/ethnicity, VA, IOP, spherical equivalent, central corneal thickness (CCT), phakic or pseudophakic status, or cataract grade among all three groups (*P* ≥ 0.064 one-way ANOVA). The average VF MD of normal, GS, and OAG subjects were -1.58 ± 1.94, -1.31 ± 3.11 and -5.36 ± 6.12 dB (*P* = 0.014). For the structural measurements, significant differences were detected in global RNFL thickness, total macula thickness, GCIPL thickness, and CDR among normal, GS, and OAG groups (*P* ≤ 0.004; Table 1).

**Table 1:** Baseline demographic information and characteristics for normal (NG), glaucoma suspect (GS), and open-angle glaucoma (OAG) groups. All values are percentages or mean ± standard deviation and median.

	NG	GS	OAG	*p-value*
	(N = 25)	(N = 16)	(N = 54)	
Age	60.6 ± 17.4	67.8 ± 10.3	67.9 ± 11.6	0.064*
Sex (Male)	8 (32.0%)	8 (50.0%)	23 (42.6%)	0.489¥
Race (White)	19 (76.0%)	12 (75.0%)	39 (72.2%)	0.437
Diabetes Mellitus	7 (28.0%)	2 (12.5%)	7 (13.0%)	0.221¥
Systemic Hypertension	15 (60.0%)	9 (56.3%)	34 (63.0%)	0.883¥
Systemic Inflammatory Disease on Immunosuppressive	5 (20.0%)	2 (12.5%)	4 (7.4%)	0.264¥
LogMAR VA	0.116 ± 0.102	0.090 ± 0.108	0.083 ± 0.110	0.457*
Spherical Equivalent (Diopters)	-1.81 ± 3.01	-1.59 ± 2.70	-1.94 ± 3.41	0.927*
IOP (mmHg)	14.7 ± 3.2	14. 3 ± 3.4	13.9 ± 3.6	0.380*
Number of IOP lowering medications	0 ± 0	0 ± 0	1.9 ± 1.4 (range 0–5)	**<0.001** *
Cup to Disc Ratio	0.33 ± 1.60	0.62 ± 0.12	0.73 ± 0.17	**<0.001** *
RNFL thickness (NG N = 13)	94.1 ± 18.7	88.2 ± 10.7	69.2 ± 14.2	**<0.001** *
Total Macula thickness (microns) (NG N = 21)	287.3 ± 18.3	281.1 ± 15.0	272.9 ± 16.7	**0.004** *
GCIPL thickness (microns) (NG N = 21)	57.80 ± 11.00	55.07 ± 5.82	50.32 ± 6.00	**<0.001** *
VF MD (dB) (NG N = 9)	-1.58 ± 1.94	-1.31 ± 3.11	-5.36 ± 6.12	**0.014** *

*One-way ANOVA

¥ Pearson Chi-square; VA = visual acuity; IOP = Intraocular Pressure; RNFL = retinal nerve fiber layer thickness; GCIPL = ganglion cell inner plexiform layer; VF MD = visual field mean deviation

**Fig 1:**
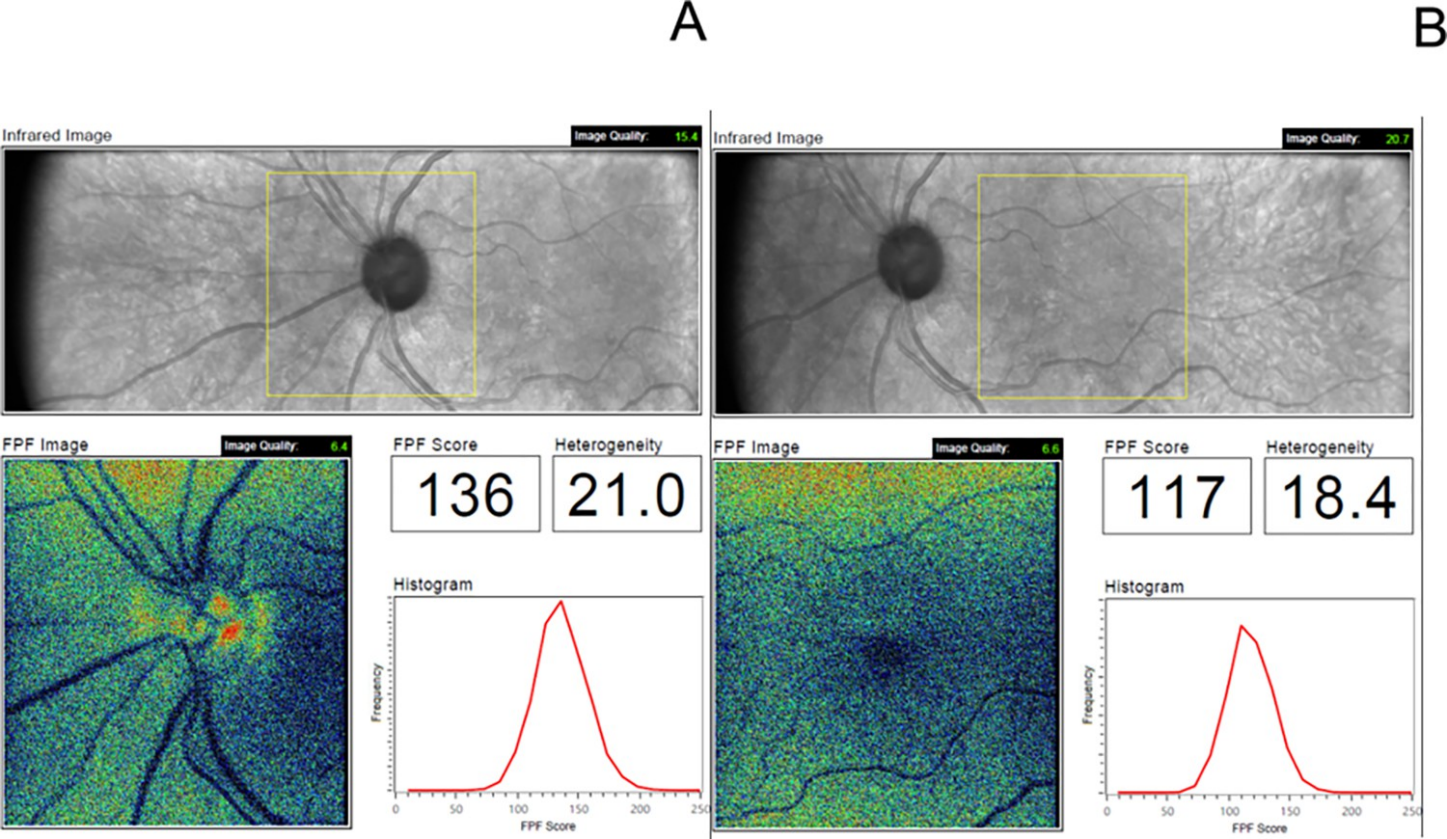
Flavoprotein fluorescence (FPF) printouts: (A) optic nerve head, (B) macula. The yellow box indicates the region where FPF is measured. In the FPF heatmap, warmer colors represent higher FPF intensities. The histogram presents the distribution of FPF intensities among all image pixels.

Fig 1 shows examples of the integrated OcuMet Clinician Report in a glaucomatous eye. Table 2 summarizes the findings for FPF scores between the groups. No significant differences were found between normal, GS, and OAG eyes in ONH or macula FPF mean scores and heterogeneity (*P* ≥ 0.163, ANOVA). Additionally, no differences were found in ONH FPF scores normalized by RNFL thickness nor on macula FPF scores normalized by temporal RNFL, macula or GCIPL thicknesses (*P* ≥ 0.062, ANOVA). We divided glaucoma patients by severity, and no significant differences were found between severity groups (*P* ≥ 0.413) or between diagnosis groups (*P* ≥ 0.173).

**Table 2:** Summary of optic nerve head and macula flavoprotein fluorescence (FPF) metrics between normal (NG), glaucoma suspect (GS), and open-angle glaucoma (OAG) group.

	NG	GS	OAG	p-value
	(N = 25)	(N = 16)	(N = 54)	
Nerve FPF	78.8 ± 33.7	91.4 ± 28.5	83.2 ± 33.8	0.492*
mean score	[74.0]	[97.5]	[80.5]	
Nerve FPF	18.4 ± 3.8	19.2 ± 4.3	20.5 ± 4.8	0.163*
heterogeneity	[18.1]	[19.5]	[20.6]	
Macula FPF	89.0 ± 27.2	93.4 ± 19.5	92.7 ± 32.9	0.854*
mean score	[87.0]	[94.5]	[93.0]	
Macula FPF	18.6 ± 3.8	17.4 ± 3.4	19.1 ± 5.0	0.415*
heterogeneity	[27.2]	[16.3]	[17.9]	
Nerve FPF mean score/	0.91 ± 0.45	1.01 ± 0.31	1.25 ± 0.57	0.062*
RNFL thickness (NG = 13)				
Macula FPF mean score/Temporal RNFL thickness (NG = 13)	0.73 ± 0.12	0.45 ± 0.12	0.85 ± 0.12	0.675*
Macula FPF mean score/Macula thickness (NG = 21)	0.31 ± 0.10	0.33 ± 0.07	0.34 ± 0.12	0.699*
Macula FPF mean score/GCIPL thickness (NG = 20)	1.70 ± 0.57	1.69 ± 0.39	1.82 ± 0.71	0.533*

*One-way ANOVA; RNFL = retinal nerve fiber layer; GCIPL = ganglion cell inner plexiform layer

Since natural lens fluorophores may impact FPF signal, we performed a subgroup analysis of only pseudophakic eyes (10 normal, 4 GS and 28 OAG eyes); no significant differences were found between groups (Table 3).

**Table 3:** Summary of optic nerve head and macula flavoprotein fluorescence (FPF) metrics between *pseudophakic* normal (NG), glaucoma suspect (GS), and open-angle glaucoma (OAG) group.

	NG	GS	OAG	p-value
	(N = 10)	(N = 4)	(N = 28)	
Nerve FPF mean score	62.5 ± 21.9	57.0 ± 17.1	64.6 ± 28.6	0.861*
Nerve FPF heterogeneity	19.9 ± 4.6	21.2 ± 7.5	21.8 ± 5.1	0.609*
Macula FPF mean score	77.3 ± 15.3	78.5 ± 27.6	78.5 ± 28.5	0.992*
Macula FPF heterogeneity	21.2 ± 4.0	19.6 ± 5.6	20.6 ± 6.0	0.883*
Nerve FPF mean score/RNFL thickness	0.7 ± 0.3	0.7 ± 0.2	1.0 ± 0.4	0.212*
Macula FPF mean score/Temporal RNFL thickness	1.3 ± 0.4	1.0 ± 0.5	1.4 ± 0.7	0.606*
Macula FPF mean score/Macula thickness	0.3 ± 0.1	0.3 ± 0.1	0.3 ± 0.1	0.842*
Macula FPF mean score/GCIPL thickness	1.4 ± 0.4	1.4 ± 0.4	1.6 ± 0.6	0.729*

*One-way ANOVA; RNFL = retinal nerve fiber layer; GCIPL = ganglion cell inner plexiform layer

Table 4 summarizes the differences in OCT biometric parameters and ONH nerve head and macula FPF metrics between the groups. Both normal eyes and glaucoma suspects showed statistically significantly thicker RNFL and GCIPL thicknesses compared to glaucomatous eyes (*P* ≤ 0.011), but no significant differences were detected between normal and glaucoma suspects (*P* ≥ 0.330). Additionally, no differences were found in ONH and macula FPF scores nor normalized FPF scores between groups (*P* ≥ 0.032, t-test with Bonferroni adjustment).

**Table 4:** Results of statistical analysis of OCT biometric parameters and optic nerve head and macula flavoprotein fluorescence (FPF) metrics between each diagnosis group.

	RNFL thickness	Nerve FPF mean score	Nerve FPF mean score/RNFL thickness	GCIPL thickness	Macula FPF mean score	Macula FPF mean score/GCIPL thickness
Normal vs. Suspect	0.330	0.207	0.490	0.348	0.551	0.852
Normal vs. Glaucoma	<0.001*	0.586	0.032	0.007*	0.605	0.309
Suspect vs. Glaucoma	<0.001*	0.346	0.042	0.011*	0.912	0.274

All values listed are P values ANOVA with Bonferroni adjustment. RNFL = retinal nerve fiber layer; GCIPL = ganglion cell inner plexiform layer

*Statistical significance after Bonferroni adjustment.

Table 5 presents the findings of univariate regression analyses between ONH and macula FPF scores, and functional and structural measurements for the OAG group. There was no significant difference in any FPF metric between the study groups in either the macula or the ONH (P ≥ 0.110).

**Table 5:** Summary of correlation (r) and univariate regression analyses results between optic nerve head and macula FPF metrics and other functional and structural clinical measurements for glaucoma group (N = 54).

	Nerve FPF mean scorer	p-value	Macula FPF mean score r	p-value
Intraocular pressure (mmHg)	0.071	0.608	0.071	0.608
Cup-to-disc ratio	-0.163	0.238	-0.148	0.285
V Visual Field Mean Deviation	0.209	0.130	0.208	0.131
V Visual Field Pattern Standard Deviation	-0.129	0.351	-0.087	0.530
Visual Field Index	0.168	0.225	0.212	0.123
Global RNFL thickness	0.243	0.169	0.216	0.124
Temporal RNFL thickness	0.080	0.571	0.032	0.819
Macula thickness	0.169	0.227	0.099	0.481
GCIPL thickness	0.223	0.108	0.131	0.349

RNFL = retinal nerve fiver layer; GCIPL = ganglion cell inner plexiform layer

## Discussion

Mitochondria are the essential cellular components for energy production, apoptosis, steroid synthesis, cellular signaling, and maintenance of homeostasis. In the context of glaucoma, mitochondrial dysfunction due to oxidative stress has been proposed as a potential biomarker [13], with FPF emerging as a non-invasive imaging technique that may be useful for assessing clinical severity, predicting outcomes, and monitoring treatment responses. Our study investigated *in vivo* mitochondrial function using a commercially available FPF imaging system across normal, GS, and OAG eyes, focusing on the ONH and macula regions. We found no significant differences in FPF scores between the studied groups.

The recognition of mitochondrial dysfunction as a contributor to glaucomatous optic neuropathy emphasizes the need for reliable biomarkers [4]. However, the absence of a normative reference dataset for FPF in healthy eyes and the variations in scanning protocol complicate its interpretation. In our study cohort, where the median age was 66.0 years, we observed a mean macula FPF of 89.0, which was higher than previous research involving normal eyes. Muste et al. [13] reported a mean macula FPF intensity of 20.0 in 228 normal eyes with a median age of 71.8 years, Chen et al. [14] found a mean macula FPF intensity of 73.0 in 151 patients with a median age of 63.5 years, and Ahsanuddin et al. [15] reported a mean macula FPF intensity of 30.62 in 21 normal eyes with a median age of 55 years (Table 6). The variations in normative data underscore the need for a standardized reference dataset, considering factors such as age and other demographic factors.

**Table 6:** Summary of studies on mitochondrial flavoprotein fluorescence (FPF).

Study	Area of interest	FPF imaging details	Eyes included	Main results
Current study	Macula and optic nerve	60° x 21° IR image	26 controls	No significant difference in FPF metrics and normalized FPF metrics between the study groups in either the macula or the ONH
		17° x 21° FPF image	16 glaucoma suspects	
			54 OAG	
Geyman et al. [9]	Macula	23° × 23° IR image	32 controls	OHT eyes had higher macular FPF (437 ± 141 vs. 327 ± 91) and macular FPF/RGC layer thickness ratios compared to control (4.8 ± 1.5 vs. 3.6 ± 1.2); OAG eyes higher FPF/RGC layer thickness ratio compared to normal (5.4 ± 2.1 vs. 3.6 ± 1.2)
		Central 13° diameter circular FPF image	38 OAG	
			6 OHT	
Zhou et al. [10]	Peripapillary	23° × 23° IR image	20 controls	FPF in the peripapillary area was higher in OAG (46.4 ± 27.9) compared to controls (28.0 ± 11.7)
		Central 13° diameter circular FPF image	50 OAG	
Muste et al. [13]	Macula	23° × 23° IR image	228 controls	Intermediate,
		Central 13° diameter circular FPF image	228 AMD	geographic atrophy, and neovascular AMD
				had higher FPF compared to controls
Chen et al. [14]	Macula	60° x 21° IR image	151 controls	Median FPF intensity and heterogeneity were higher in diabetic eyes compared to age-matched control eyes (76.0 and 0.65 vs. 71.1 and 0.5)
		19° FPF image	117 diabetics	
Ahsanuddin et al. [15]	Macula	60° x 21° IR image	21 controls	FPF intensity was significantly higher in RVO, DR, exudative AMD, and CSR compared to controls (53.80 ± 17.97; 61.75 ± 19.84; 67.47 ± 17.77; 53.80 ± 14.34; 30.62 ± 8.03, respectively)
		17° x 21° FPF image	20 RVO	
			20 DR	
			17 chronic exudative AMD	
			10 CSR	
Sun et al. [16]	Peripapillary	60° x 21° IR image	8 OAG	FPF scores were lower 1 month after intervention compared to prior (12.7 ± 11.6 vs. 10.5 ± 7.5)
		Central 19° diameter circular FPF image		

IR = infrared; OAG = open-angle glaucoma; AMD = age-related macular degeneration; RVO = retina vein occlusion; CSR = central serous retinopathy; DR = diabetic retinopathy

Geyman et al. [9] examined 32 control eyes, 38 OAG, and 16 OHT eyes, and reported both macular FPF and macular FPF/RGC layer thickness ratios were increased in OHT compared with control eyes. While OAG eyes did not exhibit a difference in macular FPF compared to controls, there was a significant difference in macular FPF/RGC layer thickness ratio. However, direct comparisons with our study findings are challenging due to differences in imaging areas and methodologies. The Geyman study was performed with a prototype device that captured a 23° × 23° IR fundus image and measured the FPF within a central 13° diameter field (Table 5). Our results are based on the commercially available OcuMet Beacon, which captures a 60° x 21° IR image and measures FPF in a 17° x 21° area.

For the optic nerve region, Sun et al. [16] reported overall ONH FPF based on a 60° IR image and 19° FPF image scores in 8 subjects with OAG before and after 1 hour of negative pressure application over the subject eyes. They reported a baseline overall score of 19.1, which did not change after 1 hour of negative pressure of intervention. Zhou et al., [10] used the investigational version of the device to evaluate 50 eyes of 30 patients with OAG and 20 normal eyes. They reported that peripapillary ONH FPF was significantly higher in OAG versus non-glaucomatous eyes. The FPF also showed a correlation to VF MD, VF PSD, and RNFL thickness [11].

The device does not measure the total number of mitochondria within a specific retinal layer; to address this limitation and the potential impact of a reduced number of mitochondria or dysfunctional mitochondria in OAG, we calculated a ratio of FPF intensity by dividing FPF scores by the global RNFL thickness, which serves as a surrogate for the total number of mitochondria in the RNFL layer, but we found no significant differences between FPF metrics among the groups, though ONH FPF mean score/RNFL thickness was found to be borderline significant. While normal and glaucoma suspects had significantly thicker RNFL and GCIPL thickness compared to glaucoma in our multiple comparison analysis, the P values were highly significant for RNFL thickness but only borderline significant for GCIPL after Bonferroni adjustment. OCT RNFL parameters have been reported to be slightly more accurate than macular parameters for detecting glaucoma [17], and the difference found in our results might be due to higher differences and accuracies for normalized FPF scores’ denominators.

In agreement with Geyman et al, [9] we found no significant association between FPF scores and IOP, macula thickness, GCIPL thickness, global and temporal RNFL thickness, VF MD, or VF PSD (Table 4). As OAG worsens and these variables worsen, the changes in FPF may be minimal due to the decreased pool of mitochondria available to fluoresce. We subdivided glaucoma into mild, moderate, and severe categories based on VF MD but found no differences between severity or diagnosis groups.

We considered potential confounding factors. The crystalline lens contains tryptophan and nontryptophan fluorophores [18], introducing a layer of complexity to FPF measurements. As the lens ages, its fluorescence intensifies, particularly in mature cataracts where there is an increase in fluorescence within the blue-green spectrum (430–480 nm). This spectrum is close to the emission region of fluorophores and can potentially introduce confounding fluorescence. While the OcuMet Beacon is designed to minimize signal contamination with narrow band-pass filters and special optical pathways, issues may arise in individuals with advanced cataracts. Despite no significant differences in visual acuity, cataract grade and phakic/pseudophakic status between the groups, a subgroup analysis focusing on pseudophakic eyes was also performed; this analysis found no significant differences in FPF scores. Still, since intraocular lenses are designed with filters that block different parts of the spectrum [19], it could affect both the OcuMet Beacon device excitation beam and the emission light received, and therefore, careful consideration is needed before integrating FPF imaging into routine clinical practice.

Our study has limitations. Our findings, derived from a relatively small cohort of normal and glaucoma suspect eyes, require validation. The demographic composition of our study, which primarily consisted of white patients (73.7%), may limit the generalizability of our findings to other populations. The cross-sectional nature of this study inherently restricts our ability to establish definitive causal relationships or track the dynamic changes of FPF over time. Eyes with OAG received IOP lowering treatment and had statistically similar mean IOP compared to normal and GS eyes, which could be a confounding factor for FPF scores. Although the impact of IOP lowering medications on FPF has not been studied, animal studies have suggested that IOP lowering agents might offer independent neuroprotective effects, possibly through mitochondria-mediated apoptosis of RGCs [20]. In addition, the possibility exists that retinal pigment melanosomes contribute to the acquired FPF signal, although prior mouse model studies have suggested that melanosomes are relatively non-fluorescent [21]. As previously discussed, there is a wide range of FPF values reported in normal eyes. Some of those differences may stem from different device versions and custom imaging areas. Prospective studies with a longitudinal approach and standardized methodology are essential for unveiling the prognostic significance of this modality and addressing these limitations.

In summary, we have performed *in vivo* measurements of ONH and macula mitochondrial function using the commercially available OcuMet Beacon system and the integrated FPF report generator in normal, GS, and OAG, and found no significant differences between the groups. Further research is needed to establish the role of FPF measurements as a diagnostic tool for glaucoma.

## Supporting information

S1 Data Data collection spreadsheet. (XLSX)
